# Preconception education program for non-invasive prenatal testing focused on interest in genetics among female university students in Japan: a quasi-experimental study comparing pre-intervention, post-intervention, and three-month follow-up results

**DOI:** 10.1186/s13690-023-01157-5

**Published:** 2023-07-27

**Authors:** Chihiro Katada, Kazutomo Ohashi, Kimie Okada, Hideaki Sawai

**Affiliations:** 1 Department of Nursing, Hyogo Medical University, Hyogo, Japan; 2 Department of Nursing, Otemae University, Osaka, Japan; 3 Course of Obstetric Nursing, Kio University, Nara, Japan; 4 Department of Obstetrics and Gynecology, Hyogo Medical University, Hyogo, Japan

**Keywords:** Decisional conflict, Non-invasive prenatal testing, Preconception education, ARCS model, Interest in genetics

## Abstract

**Background:**

Non-invasive prenatal testing (NIPT) is offered as a reproductive choice in many countries. However, pregnant women, particularly those who are primipara or lack knowledge of prenatal testing, experience difficulties understanding adequate information and making decisions on NIPT. This study developed a preconception education program about NIPT, focusing on interest in genetics, and aimed to clarify the effectiveness of the program to help women make decisions on future NIPT.

**Methods:**

This was a one-group, quasi-experimental, pre-post-test study. The study population was female undergraduate students in Japan who participated in the education program. This program included two games and was based on the Attention, Relevance, Confidence, and Satisfaction (ARCS) model, which is an instructional design that stimulates learning interest and motivation. The data of 73 pre-pregnant women who completed all three questionnaires—before, immediately after, and three months after the intervention—were analyzed to clarify the time effects. Moreover, all variables were analyzed using logistic regression analysis to investigate factors related to decisional conflict.

**Results:**

Interest in genetics, knowledge of genetics and prenatal testing, and indecisive attitudes toward NIPT significantly improved immediately after the intervention, and consequently, these changes and reduction of decisional conflict were maintained at three months. Moreover, low decisional conflict at follow-up was significantly associated with a high interest in genetics (adjusted odds ratio, 3.42).

**Conclusions:**

These findings provide preliminary evidence that this preconception education program, which focused on facilitating interest in genetics, assists pre-pregnant women to reduce decisional conflict about future NIPT.

**Trial registration:**

The trial was registered at the UMIN-CTR registry (January 16, 2023), registration number UMIN000050047.

**Table:** 

**Text box 1. Contributions to the literature**
• Pregnant women who make decisions on NIPT face the risks of experiencing decisional conflict, regretting undergoing NIPT, and developing post-partum mental distress.
• Decision-making on NIPT requires sufficient time to consider the termination of an abnormal fetus and personal values regarding ethical issues.
• This study adds to the literature by clarifying how preconception education based on the ARCS model helps pre-pregnant women in decision-making on NIPT by reducing their decisional conflict and indecisive attitudes toward NIPT and increasing their knowledge of and interest in genetics.

## Background

Non-invasive prenatal testing (NIPT), which can reliably detect fetal chromosomal abnormalities through simple blood testing, is a reproductive choice offered to women in many countries [1]. NIPT can be performed at nine to ten weeks of pregnancy, indicating that pregnant women must make decisions about whether to undergo NIPT during their early pregnancy. However, previous studies have shown that pregnant women often experienced difficulties understanding adequate information regarding NIPT [2] or decided to undergo NIPT without due consideration [2–4]; consequently, they experienced decisional conflict [5], regretted undergoing NIPT [6, 7], and developed post-partum mental distress [8].

Decisional conflict is perceived when people face difficulties in making decisions about healthcare options [9]. It is defined as the state of uncertainty regarding the preferred choice, and people with decisional conflict feel uncertain, uninformed, unclear about personal values, and unsupported in decision-making [9]. Women who made uninformed decisions on NIPT tend to experience high decisional conflict [3], and high decisional conflict regarding prenatal testing was reportedly associated with women with no children [10] and low levels of knowledge regarding testing [11–13]. These indicate that primiparas and women with low levels of knowledge may need decision-support for prenatal testing in addition to the current care provided.

Clinical practice guidelines recommend that health professionals discuss NIPT with pregnant women, regardless of them being primipara or multipara, at their initial prenatal visit [14] and emphasize the importance of women’s autonomous decision-making regarding prenatal testing [15–17]. However, health professionals must discuss numerous topics in a limited time and are thus often unable to provide women with opportunities to understand the ethical issues and autonomy related to NIPT [3]. Moreover, a previous study in Japan showed that approximately 70% of non-pregnant women knew very little about NIPT, and this lack of knowledge was associated with indecisive attitudes toward NIPT [18]. These findings indicate that most women are not ready to consider NIPT. Hence, decision-support before pregnancy is required to build a foundation that enables every woman to make decisions on future NIPT.

Preconception decision-support for NIPT needs an intervention aimed at maintaining the effectiveness of the program until pregnancy. This study employed the Attention, Relevance, Confidence, and Satisfaction (ARCS) motivation model, which has been applied in various learning contexts as an effective educational intervention to maintain learners’ self-study [19]. The ARCS model is an instructional design to enhance learning motivation [20], and programs based on the ARCS model assist learners in improving learning interest, motivation, and continuous learning [19]. Interest is one of the most important factors in education assessment [21], and high interest reportedly enhanced long-term learning motivation and an ongoing deepening and development of learners’ knowledge and value [22]. A meta-analysis of education materials based on the ARCS model showed that the Attention dimension indicated the largest effect among all the dimensions of the model, and attention was the main component of interest [23]. These indicate that enhancing interest would be necessary for preconception education. Thus, the education program based on the ARCS model focusing on enhancing interest may maintain its effectiveness. We hypothesized that this preconception program based on the ARCS model would help women, including those who lack knowledge, to maintain an interest in genetics and, consequently, make decisions on future NIPT. This study aimed to develop a preconception education program focused on facilitating interest in genetics and to clarify its effectiveness in reducing women’s decisional conflict regarding NIPT.

## Methods

### Participants

This study recruited female undergraduate students from two universities in Kobe, Japan. Eligible women were those who had never been pregnant and understood Japanese.

Participants were recruited after classes in which researchers were not involved and spontaneously enrolled in this study. All participants provided written informed consent. Ethical approval was obtained from the Hyogo University of Health Sciences (currently known as Hyogo Medical University) Ethical Review Committee, approval numbers: 16,042 and 17,014.

### Design and procedure

This study used a quasi-experimental, one-group, pretest-posttest design. A quasi-experimental study is an evaluation that aims to determine whether an intervention has the intended effects on the participants of a study [24].

### Development of the preconception education program about NIPT

**Fig. 1:**
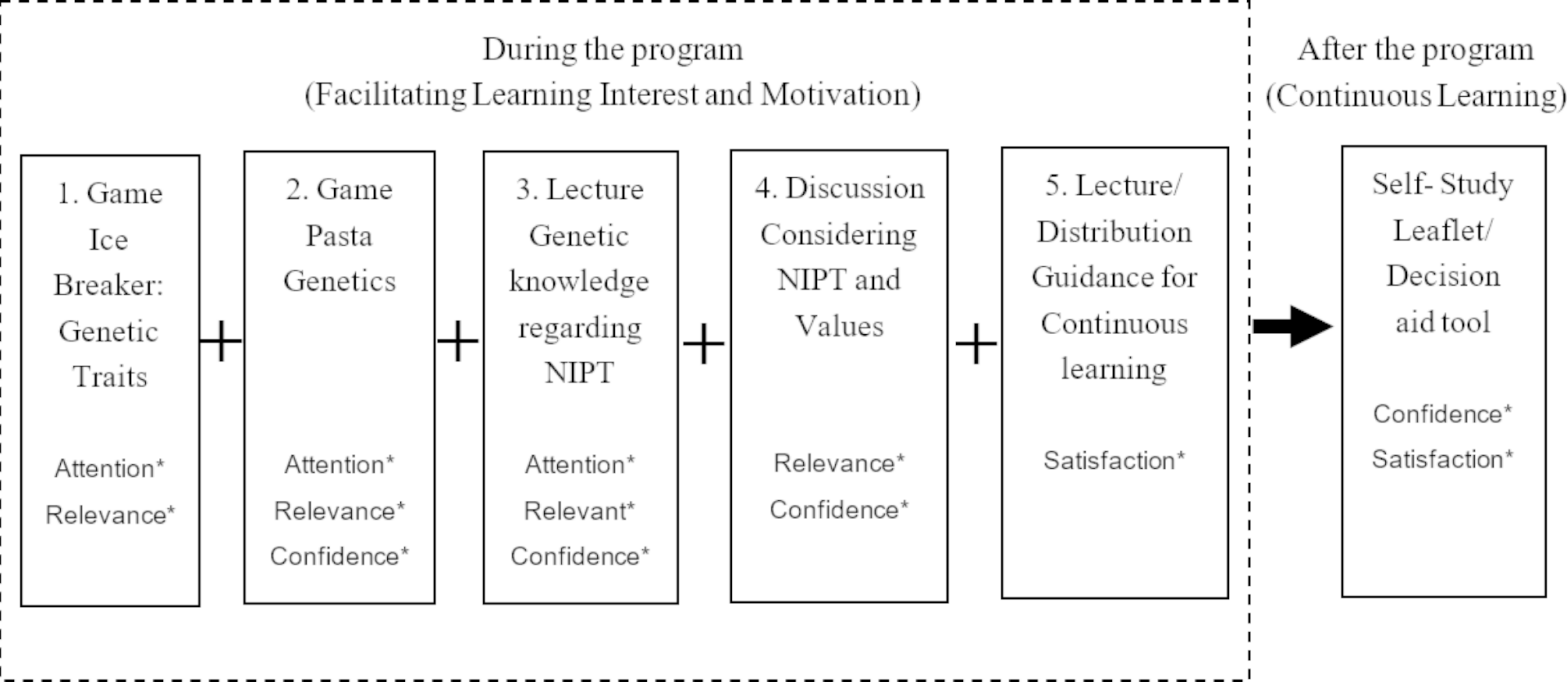
Components of the preconception education program about NIPT based on the ARCS model, Japan Note.^*^Dimensions of the ARCS model This figure was created by referring to Keller [20] NIPT: non-invasive prenatal testing; ARCS: Attention, Relevance, Confidence, and Satisfaction

This study developed an education program comprising five components (Table 1) in the form of a 90-minute workshop based on the ARCS model [20], with reference to our unpublished previous study in which 1576 women aged 20–49 were surveyed about their needs for prenatal testing, a systematic review of decision-aid tools [13], and the opinions of a genetic counselor, obstetricians, midwives, and mothers of infants with Down syndrome. Figure 1 describes the five components of the preconception education program on NIPT based on the ARCS model. The dimensions of the ARCS model are defined as follows: the Attention dimension triggers participants’ temporary interest; the Relevance dimension maintains their interest by helping them recognize the relevance of the topics being discussed; the Confidence dimension helps them build expectations for success and self-confidence to learn; and the Satisfaction dimension enhances self-learning motivation [25, 26].

**Table 1:** Components of the preconception educational program about NIPT

Components	Methods	Curricular Objectives	Materials
1. Ice Breaker: Genetic Traits	Work (Game)	Have an interest in genetics and understand differences in genetic traits of each participant	- Handout [Fig. 2] - Pencils
2. Pasta Genetics	Work (Game)	Understand Mendelian genetics	- Two cups - Colored pencils -16 pieces of pasta (eight pairs of four colors and four different shapes) that represent alleles from each grandparent
3. Genetic Knowledge/ Information regarding NIPT	Lecture	Understand genetic diversity and unique individuals Understand accuracy, safety, options about NIPT Understand ethical and social issues about NIPT Evaluate information on the Internet	- Handout
4. Considering NIPT and Values	Discussion	Think about undergoing future NIPT based on participants’ values	
5. Guidance for continuous learning	Lecture Distribution	Explain continuous learning: distribute a leaflet with additional information about prenatal testing, perceptions of parents of children with chromosomal anomalies, and support systems, and explain how to use a decision aid	- Leaflet - Ottawa Personal Decision Guide

*Note.* NIPT: non-invasive prenatal testing

**Fig. 2:**
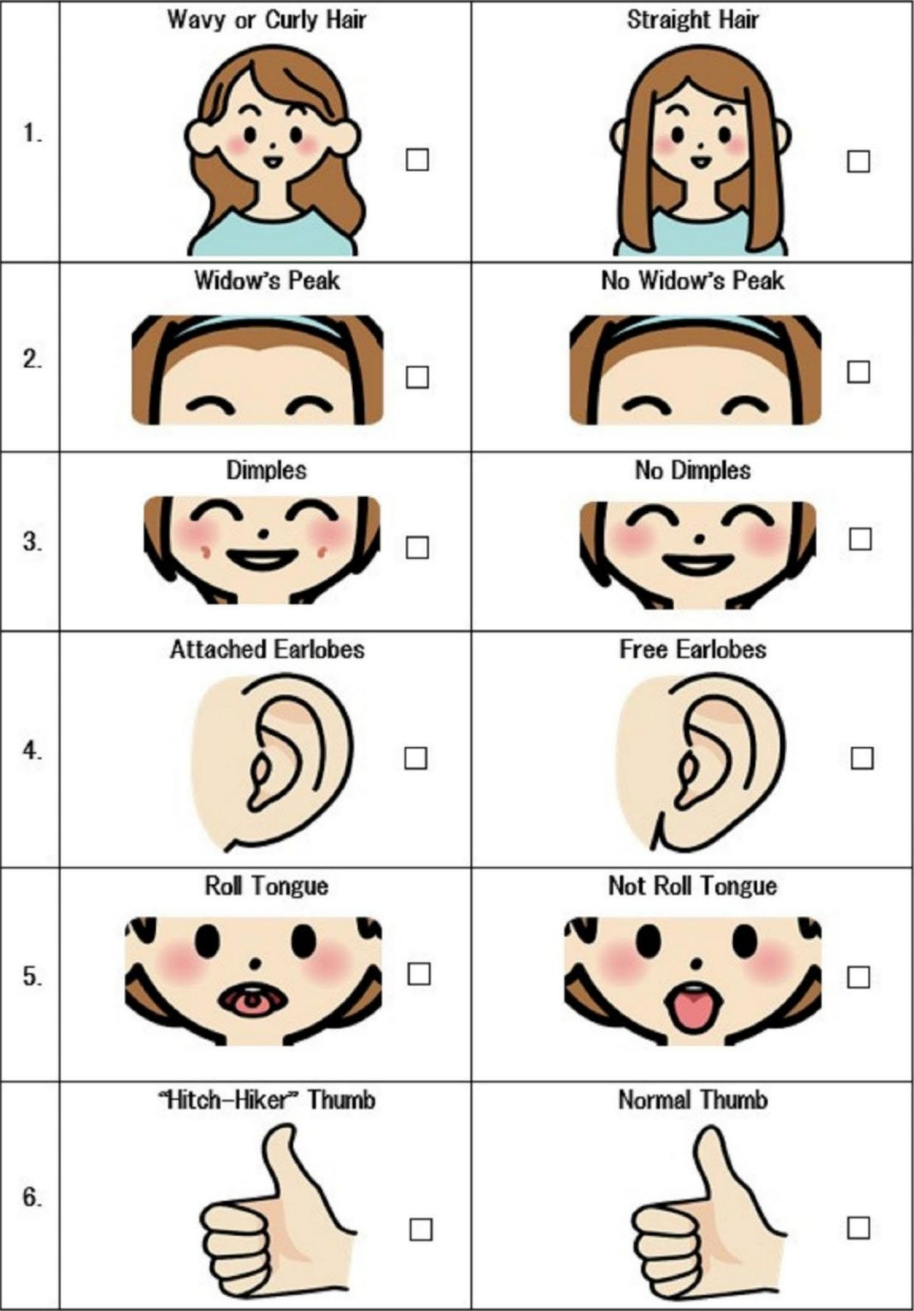
Handout to facilitate communication among participants through sharing about each other’s genetic traits

**Fig. 3:**
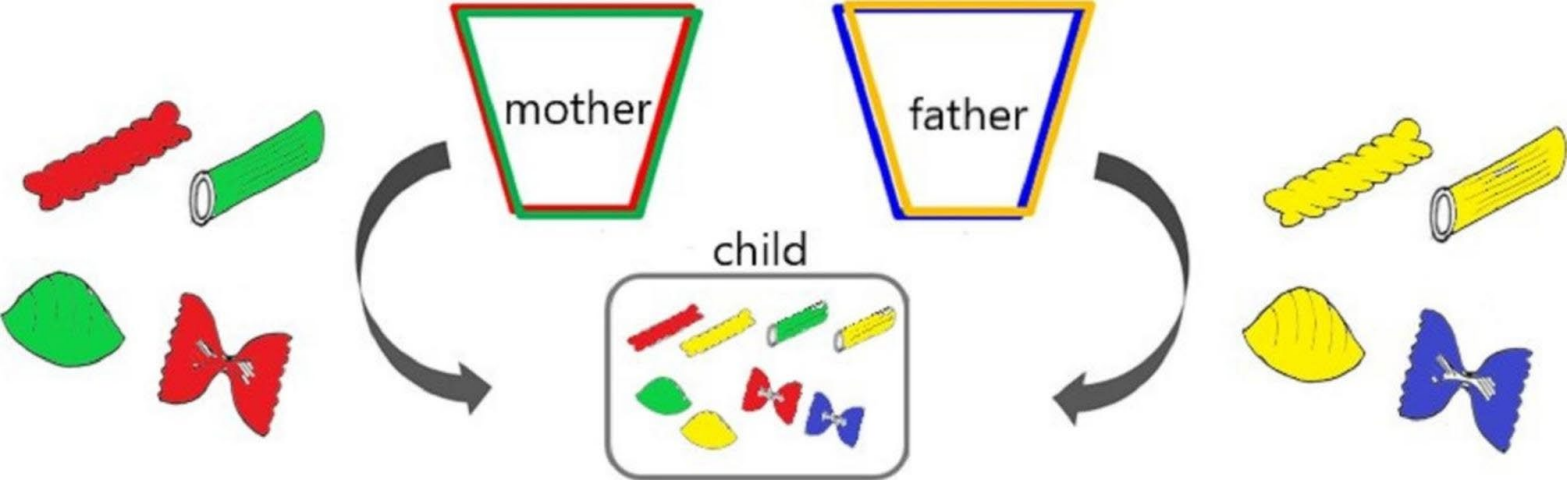
Schematic diagram of the Pasta Genetics game *Note.* This figure was created by referring to Brown and Munn [27]

First, to facilitate participants’ attention, this program employed two games about genetic traits (Fig. 2) and pasta genetics [27]. For genetic traits, participants identifying their own traits and comparing them to those of others can be an icebreaker that helps them understand the diversity and uniqueness of genetics. Pasta genetics is an educational game for teaching elementary students how genes are passed from generation to generation, using four differently shaped pasta of various colors that represent genes (Fig. 3). It is aimed at learning about the diversity and uniqueness of the combinations of the next generation’s genes. These games were employed in this study for participants to experience the joy of learning about genetics, and thereby help those who lack genetic knowledge improve their understanding of and interest in genetics. Second, to facilitate relevance and confidence, a lecture about genetic knowledge/information regarding NIPT and a discussion about NIPT considering participants’ values were provided. Making decisions about NIPT requires support for not only understanding genetic knowledge but also for discussing women’s values, which helps women make decisions and minimize future regret [28]. Group discussions involving interaction reportedly enhance interest [29]. Thus, to enhance participants’ interest in genetics and discuss NIPT based on their values, this program employed lectures about ethical and social issues regarding NIPT, such as characteristics of infants with Down syndrome, legal restrictions on the termination of a pregnancy, prejudices against people with disabilities, eugenics, autonomy, and health/information literacy. Additionally, the participants talked about the pros and cons of NIPT based on their personal values, life plans, and social/ethical issues. In the last component of the program, to facilitate self-study, continuous learning was explained and the Ottawa Personal Decision Guide [30] was distributed, which helps with decision-making. Moreover, leaflets were distributed with additional information about prenatal testing, perceptions of parents of infants with chromosomal anomalies, and support systems regarding children with abnormalities. Our previous studies reported that pre-pregnant women’s indecisive attitudes toward NIPT were associated with valuing the opinions of family members rather than their own opinions [18]. Thus, participants were recommended to discuss future NIPT with their family members using the leaflets and to reconsider the extent of the influence their family members’ opinions have on their decisions using the decision guide.

A pilot study was conducted with 18 female undergraduate students using the prototype of the program. The pilot study was assessed using the Reduced Instructional Materials Motivation Survey (RIMMS) consisting of 12 items rated on a 5-point Likert scale (1 = strongly disagree, 5 = strongly agree). The RIMMS is a short version scale based on 36 items of the original Instructional Materials Motivation Survey [31, 32], which was developed to evaluate the effect of materials using the ARCS model [33]. The RIMMS was reported to have a Cronbach’s alpha of 0.82–0.90 [33]. The mean score of the pilot study was 4.6 ± 0.6. Regarding the 90-minute duration of the program, 55.5% of the participants considered it appropriate, and 44.5% answered that it was too long. Although there was insufficient time for an in-depth discussion, further discussion with family members was recommended as part of continuous learning, and therefore, the program duration was decided to be 90 min. Furthermore, this program was modified based on a decision-aid checklist published by the International Patient Decision Aid Society to assess well-designed decision instruments [34] as well as the opinions of participants, a genetic counselor, a clinical geneticist, obstetricians, and midwives.

### Outcome measures

#### Primary outcome

Decisional conflict concerning whether to undergo NIPT was assessed by the Japanese version of the 16-item Decisional Conflict Scale (DCS) developed by O’Connor [9] and translated and validated by Arimori [35]. DCS is used to evaluate the effectiveness of decision-support interventions [36] and measures an individual’s perception when making decisions regarding feeling uncertain, uninformed, and unconfident; unclarified values; and a low level of support [9, 36]. Scores range from 0 (no decisional conflict) to 100 (extremely high level of decisional conflict). Scores exceeding 37.5 are assessed as high decisional conflict and associated with a delay or feeling unsure about implementation [35].

#### Secondary outcome

Interest in genetics was assessed using the following question: “How interested are you in genetics?” Answers were indicated as “very much,” “quite a lot,” “a little,” and “not at all.” The former two answers were classified as the high-interest group and the latter two as the low-interest group.

The level of knowledge required for deciding whether to undergo NIPT was measured using a questionnaire consisting of 20 items on knowledge of genetics and prenatal testing (Table 2). In two previous studies [37, 38], 16 items related to genetic knowledge were reported, and 15 of these 16 items were used, excluding the item “the genotype is not susceptible to human intervention” due to the development of gene therapy. Regarding knowledge of prenatal testing, five self-developed items were added. Responses were indicated as “true,” “false,” or “unsure.” One point was awarded for each correct answer and the scores were summed to assess the level of knowledge. Zero points were awarded for wrong answers and the “unsure” option. The level of knowledge was calculated by the total mean score, which ranged from 0 to 20. The Cronbach’s alpha of this scale was 0.85 in the pilot study.

**Table 2:** Questions regarding knowledge needed to decide about undergoing NIPT

**1. About knowledge of genetics**
(1) One can see a gene with the naked eye. (false)
(2) Healthy parents can have a child with a hereditary disease. (true)
(3) The onset of certain diseases is due to genes, environment, and lifestyle. (true)
(4) A gene is a disease. (false)
(5) The carrier of a disease gene may be completely healthy. (true)
(6) All serious diseases are hereditary. (false)
(7) A gene is a molecule that controls hereditary characteristics. (true)
(8) Genes are inside cells. (true)
(9) The child of a disease gene carrier is always a carrier of the same disease gene. (false)
(10) A gene is a piece of DNA. (true)
(11) A gene is a cell. (false)
(12) A gene is a part of a chromosome. (true)
(13) Different body parts include different genes. (false)
(14) Genes are bigger than chromosomes. (false)
(15) It has been estimated that a person has about 25,000 genes. (true)
**2. About knowledge of prenatal testing**
(1) Ultrasound examination can detect all fetal abnormalities. (false)
(2) It is necessary for pregnant women to undergo prenatal testing. (false)
(3) There is a risk of miscarriage in prenatal testing. (true)
(4) If a fetal abnormality is detected, a pregnant woman is able to have an abortion at any time during pregnancy. (false) ^*^
(5) There are fetal therapies for almost all fetal abnormalities. (false)

Note.^*^ In Japan, termination of a pregnancy is allowed at less than 22 weeks of pregnancy

NIPT: non-invasive prenatal testing

Indecisive attitudes toward undergoing NIPT were assessed using the following item: “Would you undergo NIPT if you were to become pregnant now?” and the possible answers were “yes,” “no,” or “unsure.” Of these, “unsure” was regarded as indecisive, and “yes” or “no” were regarded as decisive.

### Data collection

The data were collected three times: immediately before the intervention (pre-intervention), immediately after the intervention (post-intervention), and three months after the intervention (follow-up). Previous studies have shown that after learning several new words, learners tend to rapidly forget these words in less than seven days [39], and forget almost all words after three months [40]. This indicates that if participants have no continuous learning after interventions, the effects would not be sustained. Thus, we collected data three months after the intervention to assess the effects of the program and continuous learning. Online questionnaires were distributed and collected. An identification number was assigned to every participant and used throughout this study. Identification numbers were managed by a researcher who did not analyze the data. Data from pre-intervention to follow-up were linked to the identification numbers and compared. The intervention was conducted a total of 15 times, and pre-intervention questionnaires were distributed and collected in May 2017 and post-intervention questionnaires in March 2020.

### Sample size

To identify the differences in decision-making between the pre-intervention and follow-up with 80% power at a 5% level of significance, 59 female students were required. This difference was based on the study results of decisional conflict regarding prenatal testing before and after the intervention (mean ± SD; 2.19 ± 0.44 and 2.00 ± 0.52, respectively) on a scale of 0–5 [35].

### Statistical analysis

Outcomes at pre-intervention were compared with those at post-intervention and follow-up. Mean scores of decisional conflicts and level of knowledge were analyzed using repeated-measures analysis of variance (ANOVA). Regarding decisional conflict, participants whose scores exceeded 37.5 were defined as “high decisional conflict” while those whose scores were lower than 37.5 were defined as “low decisional conflict.” The dichotomous data of high decisional conflict, high interest in genetics, and indecisiveness regarding whether to undergo NIPT were analyzed using Cochran’s Q test. When significant differences were found, pairwise comparisons (post hoc test) were conducted to examine changes over time, between pre- and post-intervention, and between pre-intervention and follow-up, using paired *t*-tests or the McNemar’s Chi-squared test, adjusted using the Bonferroni correction.

Moreover, to explore the factors affecting the reduction of decisional conflict in this study’s education program, a multivariable logistic regression analysis was conducted for a low decisional conflict using the following explanatory variables: interest in genetics (high interest = 1), mean scores of knowledge of genetics and prenatal testing, and indecisive attitudes toward NIPT (indecisive = 1). Each bivariate relationship was evaluated using logistic regression. Furthermore, multivariable logistic regression analysis was conducted for all variables to explore the factors associated with a low decisional conflict at follow-up.

Data were analyzed using EZR ver. 1.32, which is a graphical user interface for R (The R Foundation for Statistical Computing, Vienna, Austria) [41]. The significance level was set at 0.05.

## Results

### Participant characteristics

A total of 241 female students were recruited and 82 spontaneously participated in the study. Of these, 73 participants (89.0%) who completed all three questionnaires—before, immediately after, and three months after the intervention—were included in the analysis. The mean number of participants for each intervention was 5.6 (range: 4–9). The characteristics of participants at pre-intervention are shown in Table 3. All participants had no history of marriage, pregnancy, fertility treatment, or NIPT by the follow-up. Participants’ decisional conflict about NIPT at pre-intervention did not show significant differences compared to their major, experience of genetic education, intention of undergoing NIPT, and interest in genetics.

**Table 3:** Participants’ characteristics and decisional conflict about NIPT pre-intervention in Japan, 2017–2020 (n = 73)

	n (%)	Decisional conflict Mean ± SD	*p* value
Age mean ± SD	21.1 ± 1.4		
Major n (%) Nursing Economics Law Rehabilitation	47 (64.4) 11 (15.1) 9 (12.3) 6 (8.2)	67.9 ± 18.8 64.3 ± 17.3 71.7 ± 18.5 66.2 ± 15.2	0.835
Experience of genetics education n (%) Yes No	57 (78.1) 16 (21.9)	75.3 ± 13.5 66.2 ± 18.5	0.109
Intention of undergoing NIPT n (%) Would want to undergo Would not want to undergo Undecided	30 (41.1) 28 (38.4) 15 (20.5)	67.7 ± 18.8 68.5 ± 16.1 66.2 ± 20.9	0.926
Interest in genetics n (%) High Low	1 (1.4) 72 (98.6)	64.0 67.8 ± 18.1	0.838

*Note.* NIPT: non-invasive prenatal testing

Table 4 compares the following parameters at pre-intervention, post-intervention, and follow-up. If these demonstrated significant differences, results of post hoc tests were shown between pre- and post-intervention, and between pre-intervention and follow-up, to clarify the time effects.

**Table 4:** Decisional conflict, knowledge, indecisive attitudes toward NIPT, and interest in genetics pre-, post-intervention, and follow-up

			Time effect *P* value	Two points’ comparison	
				(Post hoc)	*p* value
Decisional conflict					
Pre Post Follow-up	Mean ± SD	67.7 ± 18.0 41.5 ± 17.0 45.3 ± 19.0	< 0.001 ^a)^	Pre – post Pre – follow-up	< 0.001 ^b)^ < 0.001 ^b)^
High decisional conflict (37.5<) Pre Post Follow-up	n (%)	69 (94.5) 39 (53.4) 43 (58.9)	< 0.001^c)^	Pre – post Pre – follow-up	< 0.001 ^d)^ < 0.001 ^d)^
Knowledge of genetics and prenatal testing					
Pre Post Follow-up	Mean ± SD	78.6 ± 15.0 94.6 ± 5.6 90.6 ± 7.6	< 0.001^a)^	Pre – post Pre – follow-up	< 0.001 ^b)^ < 0.001 ^b)^
Indecisive attitudes toward NIPT					
Pre Post Follow-up	n (%)	15 (20.5) 5 (6.8) 4 (5.5)	< 0.005 ^c)^	Pre – post Pre – follow-up	0.034 ^d)^ 0.015 ^d)^
High interest in genetics					
Pre Post Follow-up	n (%)	1 (1.4) 64 (87.7) 47 (64.4)	< 0.001 ^c)^	Pre – post Pre – follow-up	< 0.001 ^d)^ < 0.001 ^d)^

*Note.*^a)^ Three-point comparison with repeated-measures ANOVA; ^b)^ Two-point comparison with repeated *t*-test adjusted using the Bonferroni correction; ^c)^ Three-point comparison with Cochran’s Q test; ^d)^ Two-point comparison with McNemar’s chi-squared test adjusted using the Bonferroni correction

Total sample size is 73 pre-pregnant women

NIPT: non-invasive prenatal testing; ANOVA: analysis of variance

### Decisional conflict

Decisional conflict about undergoing NIPT demonstrated a significant time effect among the three points and was significantly reduced post-intervention (mean ± SD; 41.5 ± 17.0) and at follow-up (45.3 ± 19.0) compared to pre-intervention (67.7 ± 18.0) (*p* < 0.001). Of those, the number of participants with high decisional conflict reduced significantly post-intervention (53.4%) and at follow-up (58.9%), compared to pre-intervention (94.5%) (*p* < 0.001).

### Interest in genetics

Only one participant at pre-intervention (1.4%) had an interest in genetics, but the number of participants who expressed interest increased significantly for both post-intervention (87.7%) and follow-up (64.4%), compared to pre-intervention (*p* < 0.001).

### Knowledge of genetics and prenatal testing

Knowledge of genetics and prenatal testing changed significantly among pre-intervention, post-intervention, and follow-up (*p* < 0.001). Post-hoc pairwise comparison with Bonferroni correction showed that knowledge improved significantly post-intervention and at follow-up (94.6 ± 5.6 and 90.6 ± 7.6, respectively), compared to pre-intervention (78.6 ± 15.0).

### Indecisive attitudes toward undergoing NIPT

The number of women who exhibited indecisive attitudes toward undergoing NIPT changed significantly across the three points (*p* < 0.001) and reduced significantly post-intervention and at follow-up (6.8%; post hoc *p* < 0.001; 5.5%; post hoc *p* < 0.001, respectively), compared to pre-intervention (20.5%).

### Factors associated with a low decisional conflict at follow-up

To explore factors associated with the reduction of decisional conflict, multivariable logistic regression analysis for a low decisional conflict at follow-up was conducted using high interest in genetics, knowledge, and indecisive attitudes at follow-up (Table 5). As a result, only high interest in genetics was significantly associated with a low decisional conflict (adjusted odds ratio [AOR], 3.42; 95% confidence interval [CI], 1.14–10.3). In addition, interest in genetics was not significantly associated with indecisive attitudes and knowledge.

**Table 5:** Multiple logistic regression analysis of factors related to low decisional conflictthree months after intervention

		Low decisional conflict n = 30	High decisional conflict n = 43	Crude OR (95% CI)	Adjusted OR (95% CI) ^a)^
Knowledge on genetics and prenatal testing	Mean ± SD	91.0 ± 8.0	90.3 ± 7.3	1.01 (0.95–1.08)	1.01 (0.93–1.09)
Indecisive attitudes toward NIPT	n (%)	1 (3.3)	3 (7.0)	0.46 (0.05–4.65)	0.65 (0.04–10.6)
High interest in genetics	n (%)	24 (80.0)	23 (53.5)	3.48 (1.19–10.2)^*^	3.42 (1.14–10.3)^*^

Note.^*^p < 0.05

^a)^ Included all variables

OR, odds ratio; CI, confidence interval; NIPT: non-invasive prenatal testing

## Discussion

This study developed a preconception education program focused on interest in genetics, aimed at reducing women’s decisional conflict regarding future NIPT, and demonstrated the effectiveness of the program. The program was based on the ARCS model, which is an instructional design to stimulate learning interest and motivation [25]. The results demonstrated that participants’ decisional conflict as the primary outcome was significantly reduced post-intervention (immediately after intervention) and at follow-up (three months after intervention), compared to pre-intervention, indicating that this study’s program demonstrated the sustained effects of reduction of decisional conflict. High decisional conflict has been found to be associated with uninformed decisions about NIPT [3] and decisional regret [42], and high Decisional Conflict Scale (DCS) results could accurately predict serious consequences for women in making important decisions during pregnancy [3]. This further demonstrates that preconception education aimed at reducing women’s DCS may enable them to avoid decisional regret after making decisions on NIPT. Regarding interest in genetics, only one participant (1.4%) had an interest in genetics at pre-intervention, followed by a significant increase in interest post-intervention (87.6%) and a maintained high interest at follow-up (64.4%). This indicates that although most women demonstrated a low interest in genetics pre-intervention, this program enhanced their interest. Previous studies reported that interests have the following phases: (1) interests are temporally triggered by education materials such as games, (2) these are maintained by involving activities such as games and discussion, and (3) developed and deepened by stored knowledge and value through continuous opportunities to work on the theme [43, 44]. The steps of these phases are similar to the dimensions of the ARCS model; thus, every component of this program based on the ARCS model, including games, lectures, discussion, and a leaflet and decision guide distributed as continuous learning materials, might help participants enhance and develop their interests.

Moreover, participants’ knowledge of genetics and prenatal testing was significantly enhanced through the intervention in this study. A systematic review of randomized controlled trials on decision-support interventions for NIPT showed its effectiveness in reducing decisional conflict and improving knowledge about prenatal testing among pregnant women [45], which is consistent with the present study’s results of pre-pregnant women. A high level of knowledge was reported to be significantly associated with lower scores on decisional conflict [12]; thus, this study’s education program may assist women prior to conception by reducing their decisional conflict about NIPT and improving their knowledge of genetics and prenatal testing.

Participants’ indecisive attitudes toward NIPT were also significantly reduced in this study. A systematic review reported that decision-support interventions for health treatment and screening helped people make decisions and reduce their indecisive attitudes [28], similar to the present study’s results. While people with indecisive attitudes tend to require more knowledge and information to arrive at decisions [46], adequate knowledge reportedly enabled pregnant women to make decisions regarding prenatal testing [47, 48]. Thus, knowledge of genetics and prenatal testing that improved through this study’s program seemed to help participants reduce their indecisive attitudes regarding NIPT.

Furthermore, a multiple logistic regression analysis was conducted to investigate factors associated with the reduction of decisional conflict, including interest, knowledge, and indecisive attitudes. We found that a low decisional conflict at follow-up was significantly associated with a high interest in genetics (AOR, 3.42). Although previous studies on decision-making regarding NIPT among pregnant women reported that a low decisional conflict was associated with a high level of knowledge [11–13], these results differ from the present study’s results among pre-pregnant women. This seemed to be caused by the differences between the situations of pregnant and pre-pregnant women. A study of the cognitive bias between real and hypothetical situations reported that individuals in real situations tend to be more confident in their ability to make decisions, while those in hypothetical situations tend to overestimate the support from others [49]. Decisional conflict was reportedly reduced when individuals felt certainty, clarified their values about making decisions, and felt confident and appropriately supported in their decisions [36]; thus, decisional conflict of pre-pregnant women was suggested to be different from that of pregnant women who face decision-making on NIPT. High interest, which was the only factor associated with a low decisional conflict of pre-pregnant women in this study, enhanced long-term learning motivation [50], and high learning motivation enabled high decisional self-efficacy [51]. Thus, an education program based on the ARCS model, which was effective in improving participants’ interest [23], was suggested to help participants enhance their sustained learning motivation for genetics and NIPT and consequently reduce decisional conflicts. This indicates that improving interest in genetics is necessary for preconception education about NIPT, and the ARCS model may be suitable for designing a preconception education program aimed at enhancing pre-pregnant women’s interest, thereby reducing decisional conflict.

Providing preconception education about NIPT lacks consensus in medical guidelines [14]. However, knowledge of genetics/prenatal testing, which is required when making decisions on NIPT, varies by culture/ethnicity, and East Asian [52, 53] and Latina [54] women tend to lack knowledge about genetics. This suggests that culturally tailored decision-support about NIPT is required. Moreover, concerns regarding NIPT exist around potential routinization [3] and eugenics [55] due to NIPT’s high accuracy and lack of physical burden. Thus, women require preconception education on NIPT to ensure sufficient time to consider several ethical issues regarding NIPT and to make decisions on it. This preconception education program was developed to enable easy understanding for Japanese people with low genetic literacy. The games in this program may have been too easy for women with high genetic literacy, such as Western women. Thus, healthcare providers need to consider an assessment of women’s level of genetic literacy as well as cultural and educational characteristics; moreover, the education program may require adjustments for shortening the genetic game and lengthening the discussion time.

After preconception education, women might change their decisions on NIPT according to changes in their values or circumstances. However, such changes can be considered a fundamental and essential process for clarifying their personal values and improving decision-making [56, 57] on NIPT. While decision-making on NIPT was reportedly affected by healthcare providers’ explanations [2–4, 58], social pressure [59], and the opinions of family members [60], a study showed that the majority of pregnant women who decided against NIPT had made the decisions before pre-test counseling based on their personal values [61]. This indicates that clarifying personal values is necessary for women to make decisions on NIPT. A systematic review demonstrated that decision-support tools should include methods of explicit value clarification [62]. Thus, facilitating value clarification through preconception education helps women make decisions on NIPT when becoming pregnant. Moreover, if women can understand the basic knowledge about genetics and NIPT through preconception education, the number of routine explanations during pre-test counseling can be reduced, enabling more time spent on providing additional information and individualized decision-support. This indicates that even if women’s decisions on NIPT or social conditions change over time after preconception education, pre-test counseling after conception can provide support for their decision-making.

Previous studies of pre-pregnant women reported that female high school students in Sweden [63] and college students in the United States [64] showed interest in reproductive life planning, including prenatal testing. Although clinical interventions about preconception care have been implemented for diet, folic acid, physical activity, smoking [65], and pregnancy complications [66], few studies have been conducted on NIPT. Therefore, healthcare providers providing preconception education to students, who are parent-to-be, about reproductive life planning including ethical issues related to future NIPT, through this study’s preconception education program in addition to the current standard prenatal care may be appropriate and fruitful for pre-pregnant women.

### Practice implications

Our preconception education program may improve women’s decision-making on future NIPT. In addition, the development and application of new genetic tests have progressed. Even if NIPT is routinized or targeted to include a broader range of abnormalities, the program could be adapted for women by updating the information and increasing their information literacy. The general public requires knowledge of genetics when choosing their own health or medical care, which indicates that the need for education to improve genetic literacy is increasing. This education program is one of the methods to improve the genetic literacy of the general public. Therefore, newly revised educational programs based on our program regarding other genetic testing may help people make various medical decisions. Moreover, in this study, high interest was the only factor associated with low decisional conflict among pre-pregnant women. Therefore, when developing preconception education programs, it is necessary to adopt methods that can enhance participants’ interest; for example, following the ARCS model of providing support to draw their attention temporarily, recognizing the relevance of the topics being discussed, and building their self-confidence in learning [25, 26].

### Strengths and limitations

This preliminary study developed the first preconception education program about future NIPT based on the ARCS model, which can be adapted to a diverse population because of its use of genetic games that elementary school students are able to understand. However, there are five limitations. First, the quasi-experimental, one-group, pretest-posttest design may have led to a risk of self-selection in the women voluntarily opting for preconception education. Further research using a control group with random sampling would strengthen the study design. Second, 60.3% of this study’s participants experienced high decisional conflict at follow-up. Previous studies reported that many Japanese pregnant women experienced high decisional conflict after decision-support interventions pertaining to prenatal testing [35], which is similar to our results. Further research is required to clarify the reason, including examining sociocultural characteristics for the high decisional conflict among Japanese women and strategies to reduce their conflict more effectively. Third, considering that the women in this study have to decide far in the future after receiving preconception education, further studies should clarify the best time for offering women the education program, such as during pregnancy. If the effectiveness of this program is clarified among pregnant women, it could be also made available during prenatal care. Fourth, the present study’s subjects only included female university students. Japanese women tend to value men’s opinions, and we considered it necessary for women to have the opportunity to think about this issue alone prior to conception. Thus, this study was conducted among pre-pregnant women only. However, clinical guidelines recommend that women make decisions with their partners when deciding about NIPT. Thus, further studies need to include males and couples. Moreover, our preconception program was designed to increase the understanding of and interest in genetics among women with low genetic knowledge. Many participants in our study, despite having received higher education, had low levels of knowledge about genetics. This indicates a need to improve their genetic literacy. Particularly, women without higher education might have low genetic literacy and require preconception education. Further research to evaluate the effectiveness of the program among women with various educational backgrounds is required. Fifth, this study inferred the effectiveness of the developed program in reducing decisional conflict and indecisive attitudes and improving knowledge of and interest in genetics. Therefore, further studies should add variables regarding participants’ behavior changes to assess continuous learning. Moreover, a more stratified assessment could not be performed owing to the small number of indecisive participants, and further studies that include larger sample sizes are required for a more stratified assessment of indecisive attitudes.

## Conclusions

This study developed a preconception education program based on the ARCS model that focused on interest in genetics and aimed at reducing women’s decisional conflict regarding future NIPT, and demonstrated the effectiveness of the program. The results showed an improvement in pre-pregnant women’s decisional conflict, interest in genetics, knowledge of genetics and prenatal testing, and indecisive attitudes toward NIPT through the education program. This indicates that this preconception education program may assist pre-pregnant women in reducing their decisional conflict about future NIPT. Moreover, the only factor associated with reducing decisional conflict after the intervention was interest in genetics, demonstrating that a preconception education program should include methods for enhancing participants’ interest.

## Data Availability

The datasets generated and/or analyzed during the current study are not publicly available to preserve the anonymity of study participants; they are however available from the corresponding author on reasonable request.

## Abbreviations

NIPT: Non-invasive prenatal testing
ARCS: Attention, Relevance, Confidence, and Satisfaction
RIMMS: Reduced Instructional Materials Motivation Survey
DCS: Decisional Conflict Scale
ANOVA: Analysis of variance
AOR: Adjusted odds ratio
CI: Confidence interval
